# An estimate of the prevalence of epilepsy in Sub–Saharan Africa: A systematic analysis

**DOI:** 10.7189/jogh.02.020405

**Published:** 2012-12

**Authors:** Abigail Paul, Davies Adeloye, Rhiannon George-Carey, Ivana Kolčić, Liz Grant, Kit Yee Chan

**Affiliations:** 1 Centre for Population Health Sciences and Global Health Academy, The University of Edinburgh Medical School, Edinburgh, Scotland, UK; 2 Croatian Centre for Global Health, University of Split School of Medicine, Split, Croatia; 3 Nossal Institute for Global Health, University of Melbourne, Melbourne, Australia; 4 Department of Health Policy and Management, School of Public Health, Peking University Health Science Centre, Beijing, China

## Abstract

**Background:**

Epilepsy is a leading serious neurological condition worldwide and has particularly significant physical, economic and social consequences in Sub–Saharan Africa. This paper aims to contribute to the understanding of epilepsy prevalence in this region and how this varies by age and sex so as to inform understanding of the disease characteristics as well as the development of infrastructure, services and policies.

**Methods:**

A parallel systematic analysis of Medline, Embase and Global Health returned 32 studies that satisfied pre–defined quality criteria. Relevant data was extracted, tabulated and analyzed. We modelled the available information and used the UN population figures for Africa to determine the age–specific and overall burden of epilepsy.

**Results:**

Active epilepsy was estimated to affect 4.4 million people in Sub–Saharan Africa, whilst lifetime epilepsy was estimated to affect 5.4 million. The prevalence of active epilepsy peaks in the 20–29 age group at 11.5/1000 and again in the 40–49 age group at 8.2/1000. The lowest prevalence value of 3.1/1000 is seen in the 60+ age group. This binomial pattern is also seen in both men and women, with the second peak more pronounced in women at 14.6/1000.

**Conclusion:**

The high prevalence of epilepsy, especially in young adults, has important consequences for both the workforce and community structures. An estimation of disease burden would be a beneficial outcome of further research, as would research into appropriate methods of improving health care for and tackling discrimination against people with epilepsy.

At the United Nations high–level meeting on the prevention and control of non–communicable diseases (NCDs) in 2011, there was a general consensus that NCDs are already the universal leading causes of death, and that their burden is rapidly increasing [1]. By 2030, NCDs are expected to account for the death of 52 million people [2]. Low– and middle–income countries (LMICs) that lack the resources and infrastructure to cope with the diseases are expected to bear a disproportionate amount of the disease burden [3,4]. Accurate estimates of the burden of NCDs are vital to the monitoring of diseases and policy planning, but currently lacking in many of these countries [5].

Epilepsy is the most common NCD of neurological origin that affects approximately 50 million people worldwide [1,6]. Unlike many NCDs that are related to aging, epilepsy is more prevalent in children and young adults [7]. Over 85% of epilepsy cases are found in LMICs [8], most of which occur in poor regions of Africa that have the greatest number of the world’s population under the age of 15 [9]. Epilepsy severely affects the quality of life for people. The condition is highly stigmatised because of the commonly held misconception that epilepsy is contagious and the negative meanings attached to its outward manifestation, seizures [8,10,11]. In many parts of Sub–Saharan Africa (SSA), epilepsy continues to be associated with witchcraft [11-13]. For these reasons, people with epilepsy are often socially ostracised, have reduced life chances in terms of employment and marriage [8], and are prone to having poorer self–esteem and other mental health conditions such as anxiety and depression [10]. Yet, it is known that about 70% of people with epilepsy could lead full, seizure–free lives if treated [14]. Despite this, over 90% of people in SSA with epilepsy do not receive treatment [14]. Without treatment, women with epilepsy and children are at high risk of injury especially from burns should seizures occur because of tasks as cooking over an open fire or playing near pit fires [15]. Some background details on the condition are provided in **Box 1** [15-35].

Box 1 **Aetiology and treatment of epilepsy**

Epilepsy is diagnosed when an individual experiences multiple seizures, where a seizure is disordered discharging of cerebral neurones [14]. This can occur throughout the brain, causing a generalised seizure, or be confined to one area, causing a partial seizure. These seizure types are further classified to include, amongst others, absence and atonic seizures. The likelihood of an individual to have seizures depends upon their seizure threshold, which can be lowered when the neurons develop a tendency to be hyperexcitable. This can result from a variety of risk factors [14].

**Central nervous system infections**

Epilepsy can result from CNS infection by viruses such as measles, secondary to bacterial meningitis or encephalitis, fungi and parasites, most commonly neurocysticercosis (NCC) [14]. A significant association has been recognised between cysticercosis, parasitic infection with *Taeniasolium* tapeworm larvae, and epilepsy [16,17]. The prevalence of cysticercosis is higher in developing countries where sanitation facilities are less advanced and vectors include pigs, food and faecal contamination on the water supply. Epilepsy has been shown to be more frequent in areas highly endemic for onchocerciasis [18].

Malaria is highly prevalent in many areas of SSA [19], and contracting *Plasmodium falciparum* malaria in childhood is associated with nearly a 50% chance of developing cerebral malaria and a 37.5% chance of developing epilepsy [20]. Other infectious risks include meningitis and encephalitis [14].

**Birth complications**

In Sub–Saharan Africa, two thirds of women requiring emergency obstetric care are unable to receive it [21]. Untreated adverse perinatal complications are associated with a increased risk of suffering a period of hypoxic–ischaemic encephalopathy and subsequently developing epilepsy [22,23].

***Febrile convulsions***

Children under the age of 5 may also experience febrile convulsions in the event of high fever and, whilst this is not considered epilepsy, a small number of these children go on to develop epilepsy [14]. There is a recognised association between febrile seizures and epilepsy in Sub–Saharan Africa [23,24]. The definition of epilepsy used in this paper refers to seizures unprovoked by any immediate identified cause [25], and therefore febrile convulsions themselves are excluded from calculations.

**Head injury**

A common cause of childhood epilepsy is perinatal head injury. Later in life, a traumatic head injury or surgery may also result in epilepsy. It has been noted that even mild head trauma leads to an increased risk of epilepsy and this risk is present for more than 20 years following the injury [26]. Indeed, the study found the most significant risk of developing epilepsy to be associated with war injuries. In SSA head injuries have been identified as risk factors for epilepsy [24], and in this region road traffic accidents and violent conflict are important the risk factors for head injury [15].

A number of other risk factors exist including tumours [15], vascular disease and potentially brain stressors: malnutrition and low economic conditions [27]. A proportion of people with epilepsy also have no identifiable cause, for example in Cameroon 29% had idiopathic epilepsy [28]. There are also several known genetic causes of epilepsy [29] and, although a genetic mutation cannot be identified in many individuals with epilepsy, a family history of epilepsy is often present [30].

**Treatment options**

Phenobarbitone is most commonly used antiepileptic drug in SSA [16,31] and is widely affordable [32], despite widespread poverty, as it costs only US$ 5 per person per year [33,34]. However, there is a 65–95% treatment gap [10]. In Benin, 64% of children were not being treated and 50% of those who were received treatment from traditional healers [34]. In addition, this situation, where a large number of people with epilepsy do not have access to any treatment at all, is likely to result in feelings of helplessness and frustration. Given that 70% of people will epilepsy could lead full, seizure–free lives if treated [14,35], it is important to consider the role that AEDs could play in decreasing this association and therefore decreasing the likelihood that people with epilepsy move into lower socioeconomic groups.

In the only systematic review to date based on the analysis of 28 studies published, Preux and Druet-Cabanac estimated the median prevalence of epilepsy in SSA to be 15/1000 [15], but this estimate is simply a median value from door–to–door studies in several countries. No studies have yet attempted to provide age and sex–specific prevalence of epilepsy for SSA. By way of systematic review, the aims of this study were thus: (i) to provide an updated estimate for the overall prevalence of epilepsy in SSA; (ii) to give sex and age–specific breakdown for the epilepsy prevalence for SSA; and (iii) to assess the limitations of the available data. It is hoped that the findings of this study would help improve the evidence base for informing health policy regarding epilepsy in SSA.

## METHODS

### Search strategy

Initially, a literature search was carried out based on the terms ‘epilepsy’ AND ‘Africa’ to scope out the information available. The final literature review of Medline, Embase and Global Health was undertaken on 6 March 2012. **Table 1** shows the Medline search terms. Embase and Global Health were searched with terms adjusted as necessary, as shown in Online Supplementary Document(Online Supplementary Document) and yielded 381 and 165 results respectively. Countries were included under the heading Sub–Saharan Africa based on the UN data on geographical regions and composition [36]. The searches were limited to articles published between 1980 and the search date, yielding 701 results total. This limit was put in place to ensure that the results would be relevant to the current situation in SSA. No other limits were placed on the search.

**Table 1:** Medline search strategy

1	“africa south of the sahara”/ or africa, central/ or cameroon/ or central african republic/ or chad/ or congo/ or “democratic republic of the congo”/ or equatorial guinea/ or gabon/ or africa, eastern/ or burundi/ or djibouti/ or eritrea/ or ethiopia/ or kenya/ or rwanda/ or somalia/ or sudan/ or tanzania/ or uganda/ or africa, southern/ or angola/ or botswana/ or lesotho/ or malawi/ or mozambique/ or namibia/ or south africa/ or swaziland/ or zambia/ or zimbabwe/ or africa, western/ or benin/ or burkinafaso/ or cape verde/ or cote d'ivoire/ or gambia/ or ghana/ or guinea/ or guinea-bissau/ or liberia/ or mali/ or mauritania/ or niger/ or nigeria/ or senegal/ or sierra leone/ or togo/
2	comoros/ or madagascar/ or mauritius/ or reunion/ or seychelles/
3	mayotte.mp.
4	(sao tome and principe).mp. [mp = title, abstract, original title, name of substance word, subject heading word, protocol supplementary concept, rare disease supplementary concept, unique identifier]
5	saint helena.mp.
6	prevalence/
7	prevalen*.tw.
8	incidence/
9	incidence*.tw
10	(disease adj3 burden*).tw.
11	epilep*.tw.
12	epilepsy/ or epilepsies, myoclonic/ or myoclonic epilepsies, progressive/ or lafora disease/ or merrf syndrome/ or unverricht-lundborg syndrome/ or myoclonic epilepsy, juvenile/ or epilepsies, partial/ or epilepsy, complex partial/ or epilepsy, frontal lobe/ or epilepsy, partial, motor/ or epilepsy, partial, sensory/ or epilepsy, rolandic/ or epilepsy, temporal lobe/ or epilepsy, benign neonatal/ or epilepsy, generalized/ or epilepsy, absence/ or epilepsy, tonic-clonic/ or spasms, infantile/ or epilepsy, posttraumatic/ or epilepsy, reflex/ or landau-kleffner syndrome/ or seizures/ or seizures, febrile/ or status epilepticus/ or epilepsiapartialis continua/
13	1 or 2 or 3 or 4 or 5
14	6 or 7 or 8 or 9 or 10
15	11 or 12
16	13 and 14 and 15
17	limit 20 to yr = ”1980-Current”

**Figure 1:**
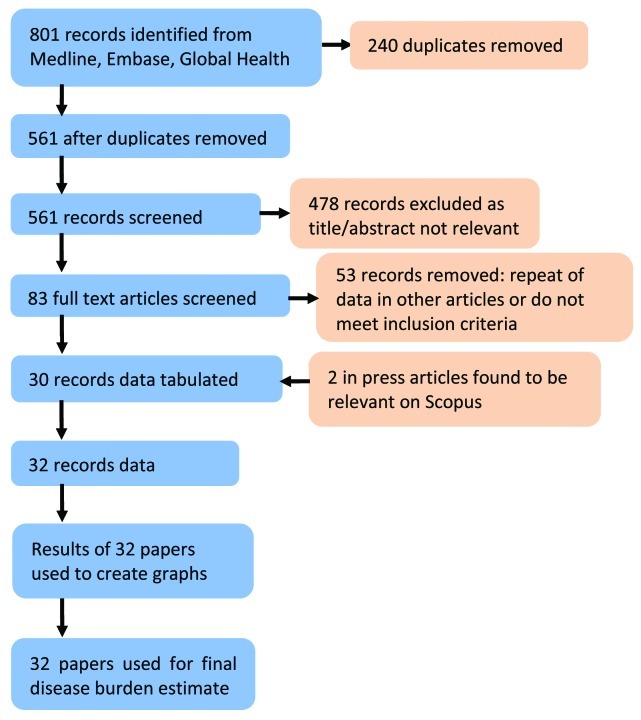
Search strategy.

Results of the 3 searches were then combined and duplicates removed, yielding 480 papers. The titles and abstracts of these 480 papers were then analysed and included if they indicated a value for prevalence in the abstract or clearly stated that a value would likely be present in the text. This generated 83 relevant papers (**Figure 1**). The full text articles were then searched to assess whether each of the studies met the full selection criteria (see **Box 2** for details of the selection criteria). Bibliographies of the selected articles and relevant review papers were searched to identify any further papers not generated by the literature searches.

Box 2 **Selection Criteria**

**Inclusion criteria**

– All age ranges

– Both sexes

– All countries in Sub–Saharan Africa

– Studies conducted to examine the epidemiology of epilepsy

– Population based studies

– Measure of disease must include a numerator and denominator

– Clear time period of study (studies were included without this factor, if all other criteria were satisfied, and the year of publication was used instead)

**Exclusion criteria**

– Health service or health record based studies

– Studies published before 1980

– Studies conducted in a population with characteristics that clearly indicated it to be unrepresentative of the national population

– Febrile convulsions and provoked seizures included in the estimation of epilepsy prevalence

**Quality criteria**

*Clear case definition and diagnosis of epilepsy* [14,25]:

*Epilepsy:* condition characterized by recurrent (two or more) epileptic seizures, unprovoked by any immediate identified cause. Multiple seizures occurring in a 24–h period are considered a single event. An episode of status epilepticus is considered a single event. Individuals who have had only febrile seizures or only neonatal seizures as herein defined are excluded from this category [25].

*‘Active’ epilepsy:* a prevalent case of active epilepsy is defined as a person with epilepsy who has had at least one epileptic seizure in the previous 5years, regardless of antiepileptic drug (AED) treatment. A case under treatment is someone with the correct diagnosis of epilepsy receiving (or having received) AEDs on prevalence day [25].

*Diagnosis:* “Diagnosis of epilepsy is essentially clinical, based on a *bona fide* history of epileptic seizures. Diagnosis should be confirmed by a health professional with expertise in epilepsy, using available medical history, seizure description, and neurologic examination. Standardized study methods should be used to obtain information about the above three diagnostic elements, and standardized criteria should be used for their interpretation. If available, EEG records and other diagnostic tools should also be used, but lack of these instruments should not preclude the diagnosis of epilepsy. EEG contributes but does not always confirm a diagnosis of epilepsy: An abnormal EEG must not be considered as a requisite for inclusion since it could be normal (or indicate nonspecific abnormalities) in epileptic subjects. On the other hand, an abnormal EEG (with epileptiform abnormalities), after an isolated seizure, could suggest classification of the seizure as epilepsy” [25].

### Data extraction and analysis

The data from 31 published articles that met the criteria were extracted and tabulated. Age and gender–specific prevalence data were extracted along with the size of the population that provided a denominator for the prevalence value. Available data regarding the types of seizures experienced by the participants were also extracted. Relevant data regarding the seizure types of those with active epilepsy were found in 15 papers and were categorised based on the International League Against Epilepsy (ILAE) classification of seizures [14,25]. For each study, mean age was calculated for each available subset of data with prevalence. If an age group had two boundaries, the median of the range was used. If an age group contained a lower age limit and older (eg, ‘60 years or more’), United Nations Population Division’s (UNPD) population estimates for the country and the year in which the study was conducted were used to estimate the mean age. Where there was an absence of a denominator, mean age was calculated for the amount of data for which there was a denominator (usually the whole study). This last rule also applies to age bands with only one given boundary.

The mean age of each data subset, its corresponding prevalence, and sample size were combined into a graph to illustrate the pattern of epilepsy prevalence across the age range of 0 to 60+. Each data point is represented by a circle proportional to the sample size. Ten–year age groups were used as they corresponded with the age groupings of the majority of the studies. The weighted mean of the prevalence for each age group and their 95% confidence interval were also calculated. The weighted mean of the prevalence for each age group was then multiplied by the corresponding population estimate by the UNPD for SSA for the year2010 [36]. Data for the year 2010 was used because we found no statistically significant changes in standardized prevalence of epilepsy over the period 1980–2012, while the largest and most reliable studies were conducted in the past decade. This provided an estimation of the number of people with active and lifetime epilepsy in SSA overall, as well as age and gender breakdown. For the data extracted on seizure classification, the total number of each seizure type found in all the relevant studies were calculated, and then divided by the total number of people with epilepsy. This provided a percentage value to represent the proportion of people with epilepsy likely to experience each type of seizure in SSA.

Subsequent to the literature search, two articles in press were found on Scopus and were included in the review. A study by Prischich et al was excluded from the final analysis because it was conducted in an area with high levels of onchocerciasis and contains outlier data that is not representative of the general population [37]. Osuntokun et al was removed as it was published in 1982 and gave no date for when the study was conducted, but this was likely before 1980 and therefore not relevant to this review [38].

## RESULTS

### Study characteristics

**Figure 2:**
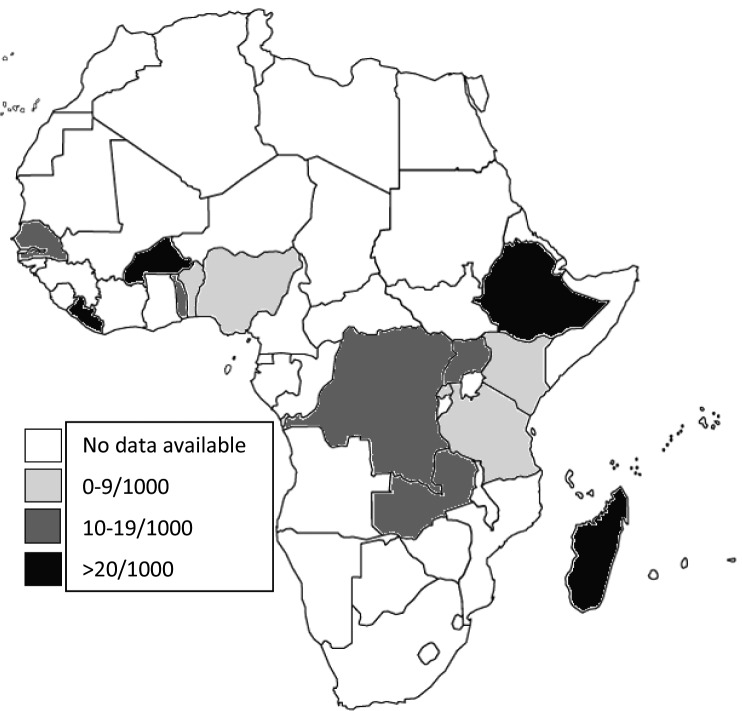
Map showing the prevalence of epilepsy by country in Sub–Saharan Africa (adapted from http://www.worldatlas.com/webimage/countrys/africa/afoutl.htm).

**Figure 3:**
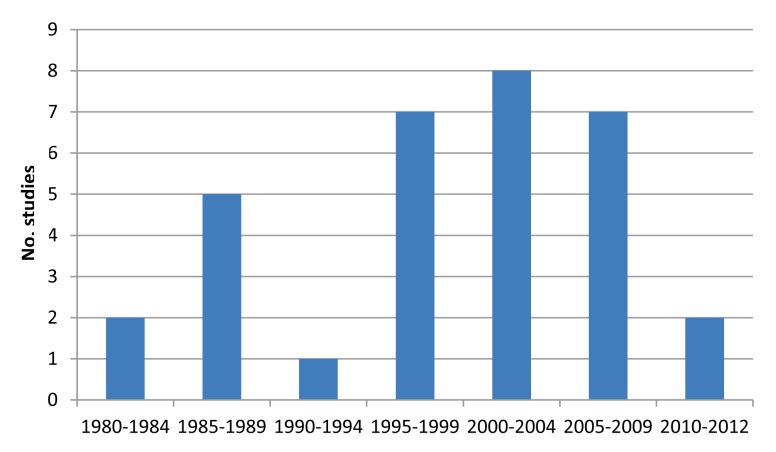
The distribution of studies according to the year of publication.

**Table 2** shows the main characteristics of the studies retained for our final analysis. They included countries from East, West, Middle and Southern Africa, and therefore can be considered reasonably representative of SSA as a whole (**Figure 2**). Only 9.4% of studies were conducted on less than 1000 individuals, 53.1% were conducted on more than 5000 individuals. There were 59.4% of the studies conducted in rural areas; as studies conducted in both rural and urban populations have shown that epilepsy is more prevalent in rural areas, the overrepresentation of rural studies in this review might represent a slight bias towards higher prevalence [30,39]. **Figure 3** shows that the studies included in the analysis were all published after 1980, with two–thirds of them published after 2000. The majority of the studies (68.8%) were door–to–door surveys, which are consistent with the ‘gold standard’ research for epilepsy prevalence estimates [40].

**Table 2:** Study characteristics (see Online Supplementary Document(Online Supplementary Document) for full table of study characteristics)

Characteristics	No. studies
**Country:**	
Benin	3
Burkina Faso	2
Cameroon	1
DRC	1
Ethiopia	1
Gabon	1
Gambia	1
Kenya	3
Liberia	1
Madagascar	1
Nigeria	3
Rwanda	1
Senegal	1
South Africa	1
Tanzania	4
Togo	3
Uganda	2
Zambia	1
**Sample size:**	
<1000	3
1000–4999	11
5000–9999	7
10 000–49 999	6
50 000–200 000	4
**Setting:**	
Rural and urban	1
Nationwide	2
Rural	19
Semirural	2
Urban	6
**Year of study:**	
Unknown	6
1980–1984	2
1985–1989	5
1990–1994	1
1995–1999	6
2000–2004	7
2005–2009	4
2010–2012	1
**Study design:**	
Cross–sectional	4
Door–to–door	22
Random sampling	3
Key informant	2
Unclear	1

### Case definitions

The ILAE Guidelines for Epidemiological Studies on Epilepsy [25] describe the diagnosis as ‘essentially clinical’. They recognise that EEG facilities are not always available, and note that EEG is not required for diagnosis. All studies included in this review comply with the ILAE guidelines as they each provide an appropriate set of criteria for clinical symptoms and diagnosis of epilepsy based upon a fitting history. Active epilepsy was diagnosed if an individual had at least one of their multiple seizures in the previous 5 years. Whilst the studies all had appropriate conditions, many had more stringent criteria for active epilepsy: 34.4% only included people who had had seizures within the last year, 15.6% in 2 years, 31.3% in 3 years and 25% in 5 years. This is likely to have led to an underestimation of the prevalence of active epilepsy. When these studies were separated based upon different definitions of active epilepsy, the median prevalence for each category was 14.4/1000 for studies requiring a seizure to have taken place in the last 1 year, 10.2 for 2 years, 38.3 for 3 years and 8.6 for 5 years. **Table 3** summarizes case definitions used in the retained studies. It shows that studies generally included a definition of active epilepsy, usually defined as a seizure within one or five years. Only five studies did not give details of the questionnaire used for case identification, while only six studies did not state that a neurologist confirmed the diagnosis.

**Table 3:** Epilepsy definitions and diagnostic criteria used by included studies

**Authors**	**Neurologist**	**EEG**	**At least one seizure in the previous year:**					**Questionnaires used**	**Sensitivity**	**Specificity**
			1	2	3	5	Lifetime			
**Case confirmed by neurologist; EEG used:**										
Balogou et al [33]	Y	Y	+				+	PAANS (2000) [41]	95%	65.10%
Balogou et al [33]	Y	Y	+					PAANS (2000) [41]	95%	65.10%
Burton et al [22]	Y	Y				+		Based on Placencia et al, 1992 [42]	98%	92%
Mung'ala-Odera et al [23]	Y	Y	+					TQQ (Durkin, et al., 1994) [43]	100%	93%
Ndoye et al [44]	Y	Y	+					GCAE (Wang et al, 2003) [45]	100%	78.50%
Osuntokun et al [38]	Y	Y		+				Based on Osuntokun et al, 1987 [38]	91%	85%
Tekle-Haimanot et al [46]	Y	Y					+	Based on Osuntokun et al, 1987 [38]	91%	85%
Kanobana et al [17]	Y	Y				+		TQQ (Durkin et al, 1994) [43]	–	–
**Neurologist; no EEG:**										
Andriantseheno et al [47]	Y	N				+		PAANS (2000) [41]	95%	65.10%
Avode et al [34]	Y	N					+	PAANS (2000) [41]	95%	65.10%
Christianson et al [48]	Y	N		+			+	TQQ (Durkin, et al, 1994) [43]	100%	93%
Coleman et al [49]	Y	N				+	+	Based on Placencia et al, 1992 [42]	98%	92%
Debrock et al [50]	Y	N					+	PAANS (1996) [51]	95%	65.10%
Dent et al [52]	Y	N				+	+	PAANS (1996) [51]	95%	65.10%
Longe et al [53]	Y	N	+					Based on Osuntokun et al, 1987 [38]	91%	85%
Ngoungou et al [32]	Y	N				+	+	PAANS (2000) [41]	95%	65.10%
Njamnshi et al [28]	Y	N					+	PAANS (2000) [41]	95%	65.10%
Rwiza et al [54]	Y	N		+				Based on Osuntokun et al, 1987 [38]	91%	85%
Winkler et al [55]	Y	N				+	+	Based on Placencia et al, 1992 [42]	98%	92%
Yemadje et al [31]	Y	N				+		PAANS (2000) [41]	95%	65.10%
Dozie et al [56]	Y	N		+				Based on Shorvon & Farmer, 1988 [57]	–	–
Dumas et al [58]	Y	N					+	No details of questionnaire	–	–
Kaamugisha & Feksi [59]	Y	N	+					No details of questionnaire	–	–
Kabore et al [60]	Y	N					+	Based on Osuntokun et al, 1987 [38]	–	–
Kaiser et al [18]	Y	N		+				History taking (epilepsy in local language)	–	–
**Case not confirmed by neurologist; no EEG:**										
Almu et al [61]	N	N	+					Based on Osuntokun et al, 1987 [38]	91%	85%
Birbeck et al [8]	N	N	+					Based on Placencia et al, 1992 [42]	98%	92%
Edwards et al [24]	Notes only	N	+					Two questions asked: convulsive seizure history, serious head injury	95%	52.30%
Goudsmit et al [62]	N	N	+					No details of questionnaire	–	–
Duggan [40]	N	N					+	No details of questionnaire	–	–
Nitiema et al [63]	N	N			+			PAANS (1996) [51]	95%	65.10%
Simms et al [39]	N	N	+					Based on Atijosan et al, 2007 [64]	99%	97%

N – no, Y – yes, PAANS – Pan African Association of Neurological Sciences, TQQ – Ten Questions' Questionnaire, GCAE – Global Campaign Against Epilepsy, WHO – World Health Organization

### Active epilepsy prevalence in the population of SSA

**Figure 4:**
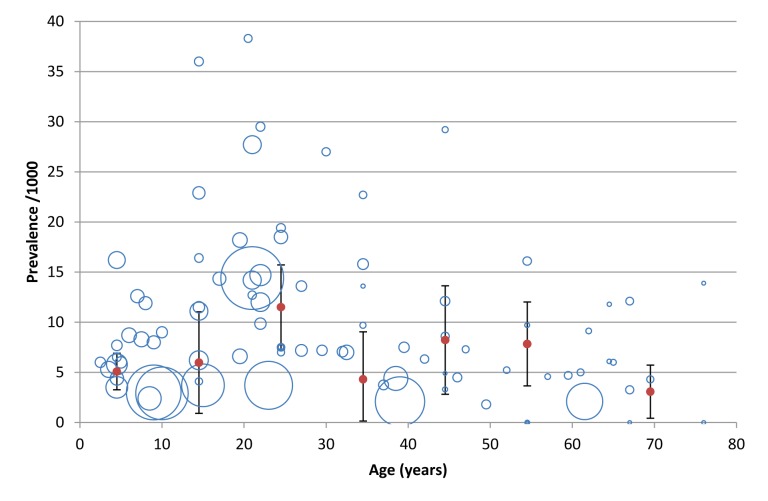
Prevalence of active epilepsy by age: the size of the bubble is determined by the size of the sample for which the prevalence was calculated in original data, while the solid red data points represent the weighted mean of the prevalence for each 10–year age group, along with the error bars representing the 95% confidence intervals.

The data points represented by a hollow blue circle in **Figure 4** show the mean age of either a subset, or the entire study population, and the corresponding prevalence for that age group. The size of the bubble is proportionate to the sample size for which the prevalence was calculated. There is a wide range of prevalence values, from 0/1000 to 36/1000. The studies with larger population base mostly yield lower prevalence values. In addition, the sample size decreases as the mean age of the study population increases. The solid pink data points represent the weighted mean of the prevalence for each 10–year age band. There is a peak of 11.6/1000 in the 20–29 age group, and a less pronounced peak of 8.2/1000 in the 40–59 age group. From the calculated weighted mean of the prevalence for each age group, the number of people in each age group with active epilepsy was calculated. In total, 5.6 million people were estimated to have active epilepsy in SSA currently. Of this, the greatest number (1.7 million) of the cases was found in the 20–29 age group (**Table 4**).

**Table 4:** Calculated weighted mean of the prevalence of active epilepsy per age group and an estimated number of cases with active epilepsy in Sub-Saharan Africa in 2010

Age group (y)	Population of SSA in 2010 (in thousands)	Number of data points	Weighted mean of the prevalence (per 1000 population) with uncertainty range (95% CI)	Estimated number of cases with active epilepsy
0–9	258 336	16	5.09 (3.27–6.91)	1 314 930
10–19	196 731	13	5.98 (0.92–11.04)	1 176 453
20–29	148 772	18	11.50 (7.29–15.71)	1 710 877
30–39	100 929	11	4.31 (0.00–9.05)	435 006
40–49	64 594	9	8.23 (2.82–13.64)	531 608
50–59	43 907	7	7.84 (3.65–12.03)	344 233
60+	43 058	12	3.08 (0.43–5.73)	132 617
Total	856 327			5 645 723

y – years, CI – confidence interval

### Lifetime epilepsy in the population of SSA

**Figure 5:**
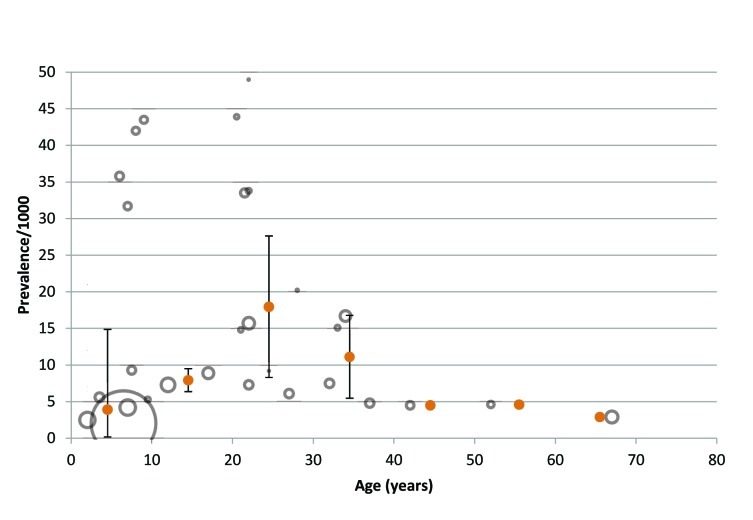
Prevalence of lifetime epilepsy by age: the size of the bubble is determined by the size of the sample for which the prevalence was calculated in original data, while the solid orange data points represent the weighted mean of the prevalence for each 10–year age group, along with the error bars representing the 95% confidence intervals.

**Figure 6:**
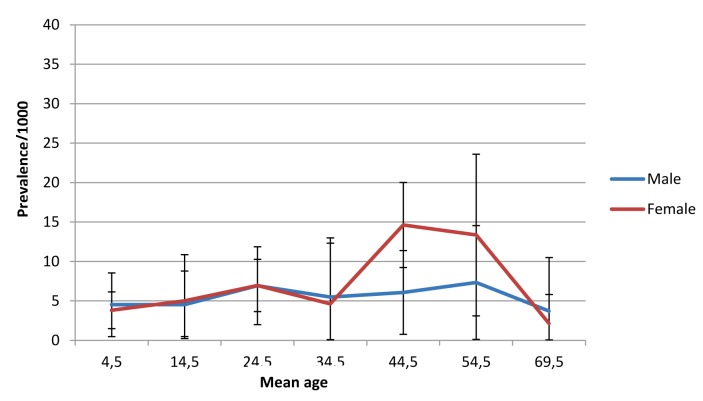
Weighted mean of the prevalence of active epilepsy per age group for men and women.

In contrast to the bimodal nature of the prevalence trend seen in active epilepsy, prevalence of lifetime epilepsy peaks in the 20–29 age group (**Figure 5**) before decreasing to a plateau in the 40–59 age group, and then further decreases in the 60+ age group. The number of people with lifetime epilepsy was calculated to be 7.0 million, with 2.7 million in the 20–29 age group (**Table 5**).The prevalence ranged between 0/1000 and 33.5/1000. Larger studies, represented by larger circles in **Figure 6**, typically reported lower prevalence values, while higher prevalence values are mostly seen in smaller study populations.

**Table 5:** Calculated weighted mean of the prevalence of lifetime epilepsy per age group and an estimated number of cases with lifetime epilepsy in Sub-Saharan Africa in 2010

Age group (y)	Population of SSA in 2010 (in thousands)	Number of data points	Weighted mean of the prevalence (per 1000 population) with uncertainty range (95% CI)	Estimated number of cases with lifetime epilepsy
0–9	258 336	10	3.91 (0.00–14.85)	1 010 093
10–19	196 731	2	7.93 (6.36–9.50)	1 560 078
20–29	148 772	10	17.96 (8.29–27.63)	2 671 943
30–39	100 929	4	11.12 (5.46–16.78)	1 122 336
40–49	64 594	1	4.51 (N/A)	291 318
50–59	43 907	1	4.65 (N/A)	204 169
60+	43 058	1	2.92 (N/A)	125 728
Total	856 327			6 985 666

y – years, CI – confidence interval, N/A – not applicable

### Sex–specific patterns of prevalence

**Figure 7:**
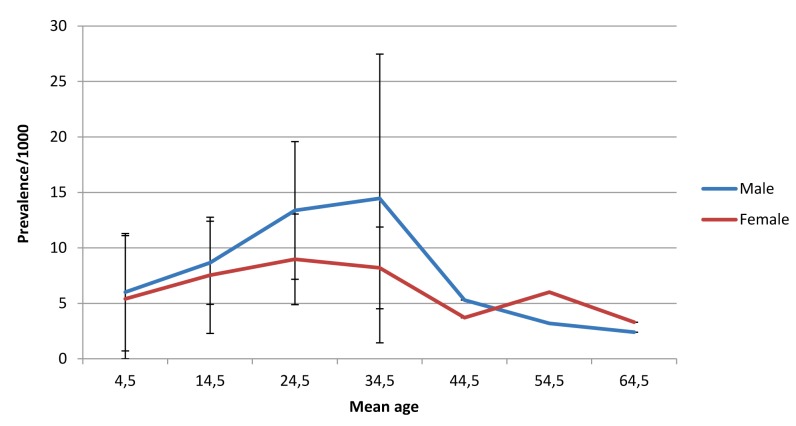
Weighted mean of the prevalence of lifetime epilepsy by sex and age group.

**Figures 6** and **7** illustrate the pattern of prevalence by sex and age. The prevalence of active epilepsy among males and females is very similar for the 0–39 age groups, though a noticeably higher prevalence of active epilepsy was observed among women relative to men in the 40–59 age group (**Figure 6**). When the prevalence trend of lifetime epilepsy is analysed by sex, the peak in the 20–39 age group is higher for men, but the second peak in the 50–59 age group is seen only in women (**Figure 7**). It is important to note that data was not included in the above graphs from all studies as not all studies provided data for separate sexes, which explains differences in comparison to **Figures 4** and **5**.

### Seizure types

Online Supplementary Document(Online Supplementary Document) shows the distribution of seizure types in the study groups for which the data were available, given that an individual may experience multiple seizure types. There was a predominance of generalised over partial seizures, with the most common generalised seizure type being tonic–clonic. Partial seizures made up 36.3% of all seizures, with a large proportion of these developing into generalised seizures. Simple partial seizures were seen more often than complex seizures.

## DISCUSSION

This paper sought to examine the prevalence trend of epilepsy in SSA by age groups and sex. The prevalence of active epilepsy can be seen to peak at two points across the lifespan: at age 20–29 and 40–59. The pattern of lifetime epilepsy also showed a peak in at age 20–29 but did not show a second peak. However, very few data were available for age 40 years and above, limiting the understanding of prevalence trends in older age groups. The peak in prevalence of active epilepsy seen in the 20–29 age group is supported by evidence in LMICs outside of SSA that saw similar peaks in young adulthood [65,66]. Whilst many studies in high–income countries have observed bimodal distribution [67], a second peak in the 40–59 age group is rarely documented in LMICs. This may be due to the fact that LMIC populations tend to have lower mean ages, and therefore older age groups are less accessible to research studies. Furthermore, as fewer data points contribute to the calculation of the weighted mean of the prevalence the older age groups, the second peak may be less reliable.

A total of 32 studies were suitable for inclusion from 18 SSA countries, resulting in a limited evidence base from which to estimate epilepsy prevalence. The searches were limited to papers published post–1980. The number of studies conducted in the past decade suggests that research interest in epilepsy in SSA has increased over the past two decades. Publication bias, where researchers conduct studies in areas where they are aware of high prevalence or high levels of risk factors (eg, onchocerciasis), might have contributed to an over estimation of the true prevalence.In contrast, stringent case definitions of active epilepsy (as having had a seizure in less than 5 years) in the majority of the studies could have resulted in an underestimation of the prevalence.

Door–to–door studies are considered to be the optimum method for obtaining disease prevalence data. However, as there were not enough studies using the standard method, we also included the studies if they were population based and did not rely on medical facilities or records, because it was assumed that a number of cases exist unknown to the medical system [68]. This included cross–sectional studies which relied on census data. One study was based in a school, and could have underestimated the epilepsy prevalence because related stigma and/or brain damage associated could have kept children out of mainstream education. Furthermore, a variety of questionnaires and data collection methods have been used, which may have varied in accuracy for identifying cases of epilepsy. This is particularly relevant in epilepsy given the wide range of seizure types, some of which present vary differently to the stereotypical tonic–clonic seizure. A larger number of the conducted studies took place in rural areas where accurate records detailing the members of the populations and where they live can be challenging. Furthermore, an address system may not be in place by which homesteads can by systematically visited. This may result in those conducting the studies missing households located in more isolated areas.

Epilepsy diagnosis can be challenging as it relies on disease history recounted by individuals rather than definitive tests, leaving ample opportunities for misinterpretation of previous events (eg, interpreting fainting as epilepsy, failure to recognise more subtle forms of epilepsy such as absence seizures) or denial of having experienced or witnessed seizures due to stigma [69]. As many people live in shared bedrooms in SSA [69], nocturnal seizures could have been witnessed and reported more accurately in history taking. As a recommendation for further epidemiological research, an increase in the number and size of population–based studies conducted in SSA would contribute to an estimation of the prevalence of greater accuracy and reliability. It would be important that further studies be conducted in accordance with the criteria recommended in **Table 6**, so as to ensure that they reap accurate results and are suitable for inclusion in future systematic reviews.

**Table 6:** Criteria for retaining identified articles on epilepsy in Africa for further analysis

Criteria	Minimum standard
Study method	A census should be conducted by the study team prior to the survey to establish the demographics of the study population. Door–to–door surveys should be conducted. Known and potential risk factors in the area should be documented.
Case definition	ILAE – active epilepsy is defined as having had two or more seizure with at least one in the previous 5 y.
Population	Studies should include all age groups and be representative of the study population. Data should be presented in 10 y age bands (0–9 y, 10–19 y, 20–29 y etc. until 60+) and be divided into males and females for each group to allow for comparisons between studies. Numerators and denominators should be available in addition to calculated prevalence.
Study size	>1000
Mode of assessment	A standardised questionnaire should be administered by workers trained in identification of a history consistent with epilepsy, including behaviours or events that are likely to be consistent with seizure activity, frequency and duration of episodes.

ILAE – International League Against Epilepsy

The stigma associated with epilepsy in SSA has significant implications for the individual. In some communities, a seizure is seen as a sign that the individual is being, or has been possessed by spirits, resulting it hem either being viewed as dangerous or as powerful by those around them and themselves [70]. Furthermore, epilepsy is considered by some groups as infectious, with 40.6% of Tanzanian individuals involved in a study believing that epilepsy was infectious and could be spread through physical contact [71]. This may lead to those with epilepsy being ostracised from their community, with a likely impact on mental health as well as physical health if stigma prevents the receipt of medical attention. The high prevalence of 11.5/1000 for epilepsy in 20–29 year-olds in SSA may affect whether women can marry and have children and whether they can fulfil their expected role in society. Furthermore, it can impact on childcare with families struggling to manage seizures or refusing to allow the child in public communal places with other family and village members. For men aged 20–29, they may find themselves excluded from manual labour or factory work because of the danger of injuries from machinery and fires. This has serious financial implications for the family and can lead to decreased socioeconomic status or even destitution [30]. The second prevalence peak affects individuals who are likely to be become grandparents and, given the epidemic of HIV in SSA increasing the number of grandparent caregivers [72], this may result in a decreased level of care for children and limited finances available to cover the basic needs of the family.

The mission statement of the Global Campaign Against Epilepsy is “To improve acceptability, treatment, services and prevention of epilepsy worldwide” [73]. This paper highlights populations that are at risk of developing epilepsy, and with whom treatment and prevention strategies should be focused. Given the wide range of environmental and genetic factors known to contribute to this prevalence, and their differing impact across time and place, it is clear that local, national and international policies are required to tackle this disease. Epilepsy care and treatment is likely to span a range of services from initial presentation of seizures to putting in place a care plan and dealing with ongoing complications, and any treatment side effects. For this to be most effective, health services need to be integrated. Given that seizures generally take place in the community and the likelihood of a seizure being witnessed by a health professional is small, it may be beneficial for health professionals to ask about seizure symptoms during consultations with general patients. Furthermore, all health professionals must be trained in the recognition of the varying presentation of this population–spanning disease. By building the effectiveness of the formal and informal health service in dealing with epilepsy, it should be expected that improved management and treatment of the seizures will reduce the fear and stigma associated with them in the community. It may be helpful to work with traditional healers who may have greater contact with people with epilepsy than health services. Epilepsy awareness and education campaigns with an aim to decrease the stigma and discrimination associated with epilepsy could provide more understanding of the nature of the disease especially in community settings. This may increase the readiness of individuals to admit to the condition and access treatment, while decreasing the negative reactions of those around them. Indeed, such campaigns also provide an opportunity to educate the population regarding safety hazards in the home for people with epilepsy, such as stoves and fires. Programmes in Togo and Kenya, combining medical treatment and psychosocial therapy, have achieved some success in reintegrating people with epilepsy into society [74,75]. The impact of any policies developed should continually be evaluated to assess whether they are achieving their goals of reducing stigma, improving treatment access and ultimately decreasing prevalence.

## CONCLUSION

Our study provided an estimate of the burden of epilepsy in Sub–Saharan Africa in the year 2010. It is likely to represent an underestimate of the true burden of the problem, because door–to–door studies are unlikely to recognize all forms of epilepsy, but rather only the most dramatic cases. More methodologically rigorous studies are needed in different parts of the SSA to improve estimates and for understanding the varying patterns of epilepsy by geographic location over time, and to inform policy more effectively. Moreover, trained health professionals and neurologists are in demand to provide more accurate diagnosis of epilepsy within this type of public health research, as well as provide care and treatment for people diagnosed. Reducing stigma could help improve diagnosis as it relies on disease history that many are ashamed to admit. Regardless of the limitations in the amount and quality of available information, this study should help inform health policy and planning in efforts to tackle this important problem in low–resource settings.
