# Supplementary material for: An estimate of the prevalence of epilepsy in Sub–Saharan Africa: A systematic analysis
Source: J Glob Health. 2012 Dec;2(2):020405. doi: 10.7189/jogh.02.020405 (PMC3529318; doi:10.7189/jogh.02.020405)
Supplement: Online Supplementary Document [file jogh-02-020405-s001.pdf]

Online Supplementary Document
Paul et al. An estimate of the prevalence of epilepsy in sub-Saharan Africa: A systematic review
Journal of Global Health 2012;2:020405

Table w1. Prevalence data extracted for epilepsy in sub-Saharan Africa

Study	Study size	Age range (years)	Mean age	Active prevalence (/1000)	Total prevalence (/1000)	Active epilepsy cases	Total epilepsy cases	Size of specific age group	Source of data
**Almu et al (2006)**	1154	0+	22	29.5	33.8	34	39		UNPD
**Andriantsehen o et al (2004)**	925	9+	30	27		25			UNPD
**Avode et al (2003)**	1400								
		<20	9.5		5.3		6	1147	Paper
		20-39	28		20.2		5	248	UNPD
**Balogou et al (2007)**	6249	0+	22	14.7	15.7		98		UNPD
**Balagou et al (2001)**	9155								
		0-9	4.5	16.2		63		3895	Paper
		10-19	14.5	22.9		48		2087	Paper
		20-29	24.5	19.4		21		1082	Paper
		30-39	34.5	22.7		18		792	Paper
		40-49	44.5	29.2		10		520	Paper
		50-59	54.5	9.7		3		309	Paper
		60-69	64.5	11.8		3		254	Paper
		70+	76	13.9		4		216	UNPD
**Birbeck et al (2004)**	55000	0+	21	14.4		793			UNPD
**Burton et al (2012)**	38523	6-14	10	2.91		112			Paper

**Christianson et al (2002)**	6692								
		2-5	3.5	5.3	5.6			3575	Paper
		6-9	7.5	8.3	9.3			3117	Paper
**Coleman et al (2002)**	16200								
		<15	8.5	2.4		18		7500	Paper
		15-24	19.5	6.6		20		3030	Paper
		25-34	29.5	7.2		11		1528	Paper
		35-44	39.5	7.5		11		1467	Paper
		45-54	49.5	1.8		2		1111	Paper
		55-64	59.5	4.7		4		851	Paper
		65-74	69.5	4.3		3		698	Paper
**Debrock et al (2000)**	3134	0+	21.5		33.5		105		Paper
**Dent et al (2005)**	4905								
		0-9	4.5	7.7		12		1554	Paper
		10-19	14.5	16.4		17		1038	Paper
		20-29	24.5	7.5		6		797	Paper
		30-39	34.5	9.7		5		516	Paper
		40-49	44.5	3.3		1		346	Paper
		50-59	54.5	0		0		304	Paper
		60-69	64.5	6.1		1		281	Paper
		70+	76	0		0		207	Paper
**Dozie (2006)**	4854	0+	22	12		58			UNPD
**Duggan (2010)**	193126	<15	6.5		2.04		395		UNPD
**Dumas et al (1989)**	5264	15-74	34		16.7		88		UNPD
**Edwards et al (2008)**	151408								
		6-12	9	3		124		41727	Paper
		13-17	15	3.7		93		25281	Paper

		18-28	23	3.7		117		31989	Paper
		29-49	39	2.1		72		34050	Paper
		50+	61.5	2.1		39		18361	Paper
**Goudsmit et al (1983)**	4436	0+	21	27.7		123			UNPD
**Kaamugisha et al (1988)**	2960	0+	19.5	18.2		54			UNPD
**Kabore et al (1996)**	1062	>15	33		15.1		16		UNPD
**Kaiser et al (1996)**	4743								
		<10	5	6		10		1793	Paper
		10-19	14.5	36		41		1128	Paper
		20-29	24.5	7		5		717	Paper
		30+	46	4.5		5		1105	Paper
**Kanobana et al (2011)**	943	0+	21	12.7	14.8	12	14		UNPD
**Longe et al (1989)**	2925								
		0-9	4.5	6.5		7		1066	Paper
		10-19	14.5	4.1		3		733	Paper
		20-29	24.5	7.5		3		398	Paper
		30-39	34.5	13.6		4		237	Paper
		40-49	44.5	4.9		1		204	Paper
		50-59	54.5	0		0		105	Paper
		60+	67	0		0		182	Paper
**Mung'ala-Odera et al (2008)**	10218								
		6	6	8.7	35.8	10	33	2940	Paper
		7	7	12.6	31.7	9	24	2493	Paper
		8	8	11.9	42	8	28	2391	Paper
		9	9	8	43.5	8	25	2394	Paper

**Ndoye et al (2004)**	4500	0+	21	14.2		64			UNPD
**Ngoungou et al (2009)**	6259	0+	24.5		9.2		58		UNPD
**Nitiema (in press)**	888	0+	20.5	38.3	43.9	34	39		UNPD
**Njamanshi et al (2007)**	1898	0+	22		49		93		UNPD
**Osuntokun et al (1987)**	18954								
		0-9	4.5	5.8		35		6003	Paper
		10-19	14.5	6.2		31		4975	Paper
		20+	38.5	4.4		35		7976	UNPD
**Rwiza et al (1992)**	18183								
		0-9	4.5	3.5		23		6522	Paper
		10-19	14.5	11.1		50		4501	Paper
		20-29	24.5	18.5		46		2482	Paper
		30-39	34.5	15.8		25		1583	Paper
		40-49	44.5	12.1		15		1243	Paper
		50-59	54.5	16.1		16		996	Paper
		60+	67	12.1		10		826	UNPD
**Simms et al (2008)**	6757								
		0-5	2.5	6		9		1520	Paper
		6-14	10	9		16		1692	Paper
		15-50	32.5	7		19		2898	Paper
		51+	61	5		3		647	UNPD
**Tekle-Haimanot et al (1990)**	60820								
		0-4	2		2.5		27	10790	Paper
		5-9	7		4.2		46	11086	Paper
		10-14	12		7.3		63	8616	Paper

		15-19	17		8.9		50	5604	Paper
		20-24	22		7.3		24	3297	Paper
		25-29	27		6.1		21	3439	Paper
		30-34	32		7.5		26	3455	Paper
		35-39	37		4.8		16	3343	Paper
		40-44	42		4.5		12	2672	Paper
		45-59	52		4.6		9	1938	Paper
		60+	67		2.9		19	6668	UNPD
**Winkler et al (2009)**	7399								
		0-9	4.5	4.4		11		2,495	Paper
		10-19	14.5	11.5		21		1,821	Paper
		20-34	27	13.6		21		1,550	Paper
		35-54	44.5	8.6		8		936	Paper
		>54	65	6		3		502	UNPD
**Yemadje et al (in press)**	13044								
		15-19	17	14.34		34		2371	Paper
		20-24	22	9.86		18		1826	Paper
		25-29	27	7.18		14		1950	Paper
		30-34	32	7.07		11		1555	Paper
		35-39	37	3.74		5		1338	Paper
		40-44	42	6.33		6		948	Paper
		45-49	47	7.3		5		685	Paper
		50-54	52	5.22		3		575	Paper
		55-59	57	4.58		2		437	Paper
		60-64	62	9.13		4		438	Paper
		65-70	67	3.26		3		921	Paper

Table w2. Prevalence data extracted for men with epilepsy in sub-Saharan Africa

Study	Study size	Age range (years)	Mean age	Active prevalence (/1000)	Total prevalence (/1000)	Active epilepsy cases	Total epilepsy cases	Size of specific age group	Source of data
**Andriantseheno et al (2004)**	407	10+	30	19.7		8			UNPD
**Avode et al (2003)**	854	5-39	18.5		3.5		3		UNPD
**Balagou et al (2007)**	3206	0+	22		17.8		57		UNPD
**Balagou et al (2001)**	4338								
		0-9	4.5	19.7		38		1932	Paper
		10-19	14.5	19.5		22		1130	Paper
		20-29	24.5	24.2		10		413	Paper
		30-39	34.5	33.5		9		269	Paper
		40-49	44.5	14.6		3		205	Paper
		50-59	54.5	15.3		2		131	Paper
		60-69	64.5	24.6		3		122	Paper
		70+	75.5	22.1		4		136	UNPD
**Burton et al (2012)**	19493	10	10	2.92		57			Paper
**Dent et al (2005)**	2194								
		0-9	4.5	11.1		8		718	Paper
		10-19	14.5	15.8		8		506	Paper
		20-29	24.5	6.3		2		315	Paper
		30-39	34.5	8.8		2		227	Paper
		40-49	44.5	0		0		141	Paper
		50-59	54.5	0		0		127	Paper
		60-69	64.5	14.5		1		130	Paper
		70+	75.5	0		0		92	UNPD

**Dozie et al (2006)**	2267	0+	22	11		25			UNPD
**Dumas et al (1987)**	2063	15-74	33.5		26.2		54		UNPD
**Edwards et al (2008)**	70024								
		6-12	9	3.4		71		21165	Paper
		13-17	15	3.4		45		13383	Paper
		18-28	23	4		55		13912	Paper
		29-49	39	2.4		33		13490	Paper
		50+	61	2.6		21		8074	UNPD
**Kaiser et al (1996)**	2466	0+	20	11.4		28			UNPD
**Longe et al (1987)**	1459								
		0-9	4.5	8.9		5		560	Paper
		10-19	14.5	5.4		2		369	Paper
		20-29	24.5	0		0		199	Paper
		30-39	34.5	0		0		96	Paper
		40-49	44.5	0		0		87	Paper
		50-59	54.5	0		0		60	Paper
		60+	67	0		0		88	UNPD
**Mung'ala-Odera et al (2008)**	5160	6-9	7.5	3.3	10.5	17	54		Paper
**Osuntokun et al (1987)**	9441								
		0-9	4.5	3.29		15		3096	Paper
		10-19	14.5	5		13		2605	Paper
		20+	38	5.4		20		3740	UNPD
**Rwiza et al (1992)**	8731								
		0-9	4.5	3		10		3297	Paper
		10-19	14.5	12.6		28		2224	Paper
		20-29	24.5	19.7		20		1015	Paper
		30-39	34.5	13.8		9		615	Paper
		40-49	44.5	7		4		570	Paper

		50-59	54.5	8		4		499	Paper
		60+	67.5	10.5		5		475	UNPD
**Simms et al (2008)**	3000	0+	21	7		21			UNPD
**Tekle-Haimanot (1990)**	29908								
		0-4	2		3.7		20	5405	Paper
		5-9	7		4.1		23	5610	Paper
		10-14	12		9.7		43	4433	Paper
		15-19	17		8.6		26	3023	Paper
		20-24	22		8.9		14	1573	Paper
		25-29	27		7.8		10	1282	Paper
		30-34	32		7.3		11	1507	Paper
		35-39	37		5.4		8	1482	Paper
		40-44	42		5.3		7	1321	Paper
		45-59	52		3.2		3	938	Paper
		60+	67		2.4		8	3334	UNPD
**Winkler et al (2009)**	3673								
		0-9	4.5	6.2		8		1296	Paper
		10-19	14.5	8.7		8		923	Paper
		20-34	27	9.6		7		728	Paper
		35-54	44.5	4.2		2		472	Paper
		55+	64.5	3.9		1		254	UNPD
**Yemadje et al (in press)**	5527	15+	33.5	9.77		54			UNPD

Table w3. Prevalence data extracted for women with epilepsy in Sub-Saharan Africa

Study	Study size	Age range (years)	Mean age	Active prevalence (/1000)	Total prevalence (/1000)	Active epilepsy cases	Total epilepsy cases	Size of specific age group	Source of data
**Andriantseh-eno et al (2004)**	518	10+	30	32.8		17			UNDP
**Avode et al (2003)**	546	5-39	18.5		14.7		8		UNDP
**Balagou et al (2007)**	3043	0+	22		13.5		41		UNDP
**Balagou et al (2001)**	4817								
		0-9	4.5	12.7		25		1963	Paper
		10-19	14.5	27.1		26		957	Paper
		20-29	24.5	16.4		11		669	Paper
		30-39	34.5	17.2		9		523	Paper
		40-49	44.5	22.2		7		315	Paper
		50-59	54.5	5.6		1		178	Paper
		60-69	64.5	0		0		132	Paper
		70+	76	0		0		80	UNDP
**Burton et al (2012)**	19030	10	10	2.89		55			Paper
**Dent et al (2005)**	2711								
		0-9	4.5	4.8		4		836	Paper
		10-19	14.5	16.9		9		532	Paper
		20-29	24.5	8.3		4		482	Paper
		30-39	34.5	10.4		3		289	Paper
		40-49	44.5	4.9		1		205	Paper
		50-59	54.5	0		0		177	Paper
		60-69	64.5	0		0		151	Paper
		70+	76	0		0		115	UNDP
**Dozie (2006)**	2587	0+	22.5	12.8		33			UNDP

**Dumas et al (1987)**	3162	15-74	34		10.8		34		UNDP
**Edwards et al (2008)**	81384								
		6-12	9	2.6		53		20562	Paper
		13-17	15	4		48		11898	Paper
		18-28	23	3.4		62		18077	Paper
		29-49	39	1.9		39		20560	Paper
		50+	61.5	1.7		18		10287	UNDP
**Kaiser et al (1996)**	2277	0+	20.5	14.5		33			UNDP
**Longe et al (1987)**	2925								
		0-9	4.5	3.9		2		506	Paper
		10-19	14.5	2.7		1		364	Paper
		20-29	24.5	15.1		3		199	Paper
		30-39	34.5	28.4		4		141	Paper
		40-49	44.5	8.5		1		117	Paper
		50-59	54.5	0		0		45	Paper
		60+	67	0		0		94	UNDP
**Mung'ala-Odera et al (2008)**	5058	6-9	7.5	3.6	11.1	18	56		Paper
**Osuntokun et al (1987)**	9513								
		0-9	4.5	6.9		20		2907	Paper
		10-19	14.5	7.6		18		2370	Paper
		20+	39	5.2		22		4236	UNDP
**Rwiza et al (1992)**	9452								
		0-9	4.5	4		13		3255	Paper
		10-19	14.5	9.7		22		2277	Paper
		20-29	24.5	17.7		26		1467	Paper

		30-39	34.5	17.2		16		932	Paper
		40-49	44.5	16.3		11		673	Paper
		50-59	54.5	22.1		12		497	Paper
		60+	68	14.2		5		351	UNDP
**Simms et al (2008)**	3751	0+	22	6.9		26			UNDP
**Tekle-Haimanot et al (1990)**	31000								
		0-4	2		1.3		7	5385	Paper
		5-9	7		4.2		23	5476	Paper
		10-14	12		5.5		23	4183	Paper
		15-19	17		9.3		24	2581	Paper
		20-24	22		5.8		10	1724	Paper
		25-29	27		5.1		11	2157	Paper
		30-34	32		7.7		15	1948	Paper
		35-39	37		4.3		8	1861	Paper
		40-44	42		3.7		5	1351	Paper
		45-59	52		6		6	1000	Paper
		60+	67		3.3		11	3334	UNDP
**Winkler et al (2009)**	3631								
		0-9	4.5	2.5		3		1,199	Paper
		10-19	14.5	14.5		13		898	Paper
		20-34	27	17		14		822	Paper
		35-54	44.5	12.9		6		464	Paper
		55+	65	8.1		2		248	UNDP
**Yemadje et al (in press)**	7517	15+	34.5	6.79		51			UNDP

Table w4. Calculated weighted mean of the prevalence of active epilepsy per age group for men and women

Men					Women				
**Age range**	**Mean age**	**Number of data points**	**Weighted mean of the prevalence**	**95% CI**	**Age range**	**Mean age**	**Number of data points**	**Weighted mean of the prevalence**	**95% CI**
0-9	4.5	8	4.52	4.03819	0-9	4.5	8	3.82	2.32785
10-19..	14.5	8	4.52	4.27653	10-19..	14.5	8	5	5.87015
20-29	24.5	9	6.94	4.93464	20-29	24.5	9	6.97	3.30833
30-39	34.5	8	5.501	7.49476	30-39	34.5	8	4.64	7.691
40-49	44.5	5	6.08	5.3077	40-49	44.5	5	14.63	5.3883
50-59	54.5	4	7.34	7.20953	50-59	54.5	4	13.36	10.2461
60+	69.5	8	3.72	6.78767	60+	69.5	8	2.14	3.68672

Table w5. Lower limit, mean and upper limits of the estimations of the number of women with active epilepsy in Sub-Saharan Africa

**Age range**	**Lower limit**	**Mean**	**Upper limit**	**Total population (thousands)**
0-9	154687	**396008**	637329	103667
10-19.	0	**388830**	845328	77766
20-29	204654	**389560**	574466	55891
30-39	0	**173457**	460970	37383
40-49	239730	**379502**	519275	25940
50-59	54920	**235630**	416341	17637
60+	0	**37437**	101933	17494
Total	653991	**2000424**	3555642	335778

Table w6. Lower limit, mean and upper limits of the estimations of the number of men with active epilepsy in Sub-Saharan Africa

Age range	Lower limit	Mean	Upper limit	Total population (thousands)
0-9	50920	**477692**	904464	105684
10-19.	19178	**356031**	692885	78768
20-29	112122	**388022**	663923	55911
30-39	0	**203323**	480336	36961
40-49	19167	**150893**	282620	24818
50-59	2142	**120479**	238816	16414
60+	0	**55023**	155419	14791
Total	203529	**1751463**	3418463	333347

Table w7. Calculated weighted mean of the prevalence of lifetime epilepsy per age group for men and women

**Men**					**Women**				
**Age range**	**Mean age**	**Number of data points**	**Weighted mean of the prevalence**	**95% CI**	**Age range**	**Mean age**	**Number of data points**	**Weighted mean of the prevalence**	**95% CI**
0-9	4.5	3	6.01	5.28827	0-9	4.5	3	5.41	5.69664
10-19..	14.5	3	8.66	3.74337	10-19..	14.5	3	7.53	5.23147
20-29	24.5	3	13.38	6.20518	20-29	24.5	3	8.97	4.08532
30-39	34.5	3	14.46	13.01291	30-39	34.5	3	8.2	3.67896
40-49	44.5	1	5.3	0	40-49	44.5	1	3.7	0
50-59	54.5	1	3.2	0	50-59	54.5	1	6	0
60+	64.5	1	2.4	0	60+	64.5	1	3.3	0

Table w8. Lower limit, mean and upper limits of the estimations of the number of women with lifetime epilepsy in Sub-Saharan Africa

**Age range**	**Lower limit**	**Mean**	**Upper limit**	**Total population (thousands)**
0-9	0	**560839**	1151392	103667
10-19.	178748	**585578**	992409	77766
20-29	273010	**501342**	729675	55891
30-39	169010	**306541**	44071	37383
40-49	-	**95978**	-	25940
50-59	-	**105822**	-	17637
60+	-	**57730**	-	17494
Total	620768	**2213830**	2917547	335778

Table w9. Lower limit, mean and upper limits of the estimations of the number of men with lifetime epilepsy in Sub-Saharan Africa

Age range	Lower limit	Mean	Upper limit	Total population (thousands)
0-9	76275	**635161**	1194046	105684
10-19.	387273	**682131**	976989	78768
20-29	401151	**748089**	1095027	55911
30-39	53486	**534456**	1015426	36961
40-49	-	**131535**	-	24818
50-59	-	**52525**	-	16414
60+	-	**35498**	-	14791
Total	918185	**2819395**	4281488	333347

Table w10. Percentage of people with epilepsy who experience each seizure type

**Generalised**	56.7%
Tonic	0.4%
Tonic clonic	32.7%
Absence	0.9%
Myoclonic	0.3%
Atonic	1.1%
**Partial**	36.3%
Simple	8.9%
Complex	4.9%
Secondary generalisation	13.8%
Unclassified to generalised or partial	7.1%
Unclassified to subtype	36.9%
